# Cutaneous Effects of In Utero and Lactational Exposure of C57BL/6J Mice to 2,3,7,8-Tetrachlorodibenzo-*p*-dioxin

**DOI:** 10.3390/toxics9080192

**Published:** 2021-08-20

**Authors:** Jyoti Bhuju, Kristin M. Olesen, Clarisse S. Muenyi, Tejesh S. Patel, Robert W. Read, Lauren Thompson, Omar Skalli, Qi Zheng, Elizabeth A. Grice, Carrie Hayes Sutter, Thomas R. Sutter

**Affiliations:** 1 Department of Biological Sciences, University of Memphis, Memphis, TN 38152, USA; jbhuju@memphis.edu (J.B.); kristin.m.olesen@gmail.com (K.M.O.); rwread1@outlook.com (R.W.R.); oskalli@memphis.edu (O.S.); csutter@memphis.edu (C.H.S.); 2 Department of Surgery, University of Tennessee Health Sciences Center, Memphis, TN 38104, USA; cmuenyi1@uthsc.edu; 3 Kaplan-Amonette Department of Dermatology, University of Tennessee Health Sciences Center, Memphis, TN 38104, USA; tpatel3@uthsc.edu; 4 Integrated Microscopy Center, University of Memphis, Memphis, TN 38152, USA; lthmps10@memphis.edu; 5 Department of Dermatology, Perelman School of Medicine, University of Pennsylvania, Philadelphia, PA 19104, USA; zhengqi@pennmedicine.upenn.edu (Q.Z.); egrice@pennmedicine.upenn.edu (E.A.G.); 6 W. Harry Feinstone Center for Genomic Research, University of Memphis, Memphis, TN 38152, USA

**Keywords:** TCDD: 2,3,7,8-tetrachlorodibenzo-*p*-dioxin, dioxin, AHR: aryl hydrocarbon receptor, skin, epidermis, atopic dermatitis, chloracne, sebaceous gland, development, in utero

## Abstract

To determine the cutaneous effects of in utero and lactational exposure to the AHR ligand 2,3,7,8-tetrachlorodibenzo-*p*-dioxin (TCDD), pregnant C57BL/6J mice were exposed by gavage to a vehicle or 5 μg TCDD/kg body weight at embryonic day 12 and epidermal barrier formation and function were studied in their offspring from postnatal day 1 (P1) through adulthood. TCDD-exposed pups were born with acanthosis. This effect was AHR-dependent and subsided by P6 with no evidence of subsequent inflammatory dermatitis. The challenge of adult mice with MC903 showed similar inflammatory responses in control and treated animals, indicating no long-term immunosuppression to this chemical. Chloracne-like sebaceous gland hypoplasia and cyst formation were observed in TCDD-exposed P21 mice, with concomitant microbiome dysbiosis. These effects were reversed by P35. CYP1A1 and CYP1B1 expression in the skin was increased in the exposed mice until P21, then declined. Both CYP proteins co-localized with LRIG1-expressing progenitor cells at the infundibulum. CYP1B1 protein also co-localized with a second stem cell niche in the isthmus. These results indicate that this exposure to TCDD causes a chloracne-like effect without inflammation. Transient activation of the AhR, due to the shorter half-life of TCDD in mice, likely contributes to the reversibility of these effects.

## 1. Introduction

Early-life exposures to environmental pollutants can impact development and lead to pathologies later in life, such as obesity, diabetes, cardiovascular, neurological, and reproductive dysfunction [1,2,3,4]. In particular, persistent organic pollutants (POPs) that are extremely resistant to degradation bioaccumulate in animals and humans, causing long-term toxic effects [5]. Common POPs include dioxins and halogenated dioxin-like compounds (DLCs) that are produced during industrial processes, including manufacturing of herbicides and pesticides, incineration, and bleaching of pulp and papers. 2,3,7,8-Tetrachlorodibenzo-*p*-dioxin (TCDD) is the prototypical and most potent compound in the family of DLCs [6] and mediates its toxic effects through the activation of the aryl hydrocarbon receptor (AHR) [7]. In utero, human fetuses are exposed to maternal dioxins through the placenta [8]. Additionally, because dioxins accumulate in fat tissue due to their lipophilic nature, breast milk is an additional and significant source of exposure for breastfed newborns [9,10].

The most significant clinical cutaneous effect of TCDD toxicity in humans is the skin condition called chloracne that develops within days following intensive exposures [11]. Chloracne results in skin lesions characterized by epithelial hyperplasia and hyperkeratosis of the interfollicular epidermis, hyperkeratosis of the hair follicle, especially at the infundibulum, and squamous metaplasia of the sebaceous glands, resulting in keratinaceous comedones and cysts, more recently characterized as hamartomas [12]. Except for hairless mice, which develop epidermal hyperplasia, involuted sebaceous glands, and dermal cysts following topical exposure to TCDD, most common research animals, including haired rodents, are mostly described as refractory to TCDD-induced dermatitis [13,14]. However, a later study reported that a two-year treatment of haired B6C3F1 mice with a relatively high-dose of the DLC, 3,3′,4,4′-tetrachlorazobenzene, results in chloracne-like skin lesions [15]. More recently, it was reported that topical application of AHR agonists, TCDD and β-napthoflavone (BNF), on C57BL/6 mice results in atrophy of the sebaceous glands without the development of cutaneous lesions as seen in chloracne [16]. In contrast, transgenic mice expressing a constitutively active AHR (AHR-CA) in the epidermis develop a phenotype that resembles atopic dermatitis (AD), including skin lesions with itching, skin inflammation, and immunological imbalance [17,18]. These studies suggest that systemic versus local, as well as the timing and duration of activation of AHR are important determinants of the effects on the skin.

The mechanism of toxicity of environmental pollutants on the skin is not completely understood. However, several studies demonstrate that AHR activation is one factor associated with a disrupted barrier, inflammatory responses, oxidative stress, and microbiome alterations [19]. Activation of the AHR increases the expression of pro-inflammatory and inflammatory cytokines that may aggravate allergic skin diseases [20,21,22]. In fact, the AHR is highly expressed in skin lesions of patients with AD and psoriasis [20,23]. Inflammatory skin conditions such as AD and psoriasis are also often associated with a defective barrier [24] and altered levels of oxidative stress [25,26]. As previously reported, AHR ligands, such as TCDD, promote differentiation in keratinocytes by altering metabolic processes to decrease glycolytic flux while increasing reactive oxygen species (ROS) [27,28].

Challenging developing embryos during organogenesis results in structural and functional defects [29,30]. In utero exposure to TCDD results in defects in several organs [31,32,33,34]. Prenatal exposure of the developing skin to TCDD in C57BL/6J mice alters the formation of the epidermal permeability barrier (EPB). The exposed embryos show accelerated development of the EPB [35,36]. At birth, exposed pups demonstrate epidermal acanthosis, hyperkeratosis, and defective barrier with leaky tight junctions [36]. Here, we investigate the developmental effects of TCDD on barrier formation and skin health, extending these observations from P1 to adult mice. The goal of this study is to determine the effects of in utero and lactational exposure to TCDD on murine skin barrier development and susceptibility to developing cutaneous disease later in life.

## 2. Materials and Methods

### 2.1. Animals and Treatment

*Ahr* null mice, B6.129-*Ahr*^tm1Bra^/J [37], and time-mated C57BL/6J mice were purchased from Jackson Laboratory (Bar Harbor, ME, USA). B6.129-*Ahr*^tm1Bra^/J mice were crossed with C57BL6/J mice and maintained for more than 10 generations in our facility. Heterozygous animals from different litters were crossed and homozygotes (*Ahr*+/+ and *Ahr*−/−) were used for the experiments. The day after a vaginal plug was observed was considered embryonic day 1 (E1). Dams received a Teklad Global 18% Protein Rodent Diet 2018 until E9 after which they were switched to a Teklad Global 16% Protein Rodent Diet 2016 (Harlan Teklad, Madison, WI, USA). Animals were housed in clear disposable plastic cages pre-bedded with ALPHA-dri (M-BTM-AD, Innovive LLC, San Diego, CA, USA) and were maintained in a 12:12-h light:dark cycle in a temperature- (22 °C ± 2 °C) and humidity- (35% ± 4%) controlled room. All animal research protocols were approved by the University of Memphis Institutional Animal Care and Use Committee and followed all guidelines and regulations. All of the animals were treated humanely. Due consideration was given to alleviate any distress and discomfort.

Treatments were administered as described previously [36]. Briefly, dams were weighed on E12 and randomly distributed into groups to receive either corn-oil (vehicle control) or a single dose of 5 or 10 µg/kg body weight TCDD in 100 µL corn-oil by oral gavage. For studies of pups past postnatal day 1 (P1), dams were treated with 5 µg/kg bw TCDD because 10 µg/kg bw TCDD impairs mammary development and lactation in the dams, ultimately affecting the survival of the pups [38]. Pups were harvested at P1, P6, P13, P21, P35, and P70. Pups remained with the mothers until weaning at P21, after which they were housed in same-sex groups of up to 5 per cage. To minimize litter bias, no more than one pup from one litter was assigned to the same timed endpoint, except for the histological and RNA endpoints at P35 and P70, where no more than two pups from one litter were assigned.

To induce AD-like pathology, adult animals exposed to either corn-oil or TCDD during development were treated with the vitamin D analog, MC903 (Calcipotriol hydrate, Sigma-Aldrich, St. Louis, MO, USA). Briefly, 2 nmol of MC903, dissolved in 25 µL of ethanol, was applied to the left ears of the mice (12.5 µL on the inside and 12.5 µL on the outside) once a day for 14 days. On the right ears, ethanol was similarly applied as a vehicle control. Ear thickening was measured with a micrometer (Mitutoyo, Aurora, IL, SUA) at indicated times.

### 2.2. Skin Permeability Assay

Barrier formation was determined using the β-galactosidase substrate 5-bromo-4-chloro-3-indolyl β-D-galactopyranoside (X-gal) as described previously [39]. Briefly, the embryos were incubated in the X-gal reaction mixture for 18 h at 30 °C, then fixed in 10% formalin at 4 °C for 24 h before transferring to 70% alcohol. Because of endogenous β-galactosidase activity in the skin at low pH, mice will turn blue following absorption of X-gal if the EPB has not formed. The embryos were photographed and quantified as previously described [35,36].

### 2.3. Transepidermal Water Loss (TEWL)

TEWL was measured on the crown of the head of the animals using the Delfin VapoMeter with a 4.5-mm nail adapter attached (Delfin Technologies Ltd., Stamford, CT, USA).

### 2.4. Measurement of Grooming Duration and Scratching Frequency

Individual mice were separated into fresh cages. After a brief acclimation period, the animals were observed for grooming behavior and scratching frequency per a 10-min period. The use of the forepaw or mouth was classified as grooming, whereas the use of the hind paw was counted as scratching.

### 2.5. Histological and Immunohistochemical Analyses

Skin samples from the dorsal back were fixed in 10% neutral buffered formalin and either embedded in paraffin or frozen in optimal cutting temperature (OCT) compound (Tissue-Tek; Sakura Finetek USA, Torrance, CA, USA). For the preparation of frozen tissues, tissues were fixed in 10% formalin for 24 h and then immersed in 20% sucrose solution for an additional 24 h at 4 °C before embedding in OCT.

For histological analysis, paraffin-embedded samples were cut into 5 µm sections, stained with hematoxylin and eosin (H&E), and visualized using an Olympus BX63 microscope (Olympus, Center Valley, PA, USA). Epidermal thickness was measured on photographs using ImageJ (National Institutes of Health, Bethesda, MD, USA).

Oil Red O for lipid staining of sebaceous glands was performed on frozen tissue. 10 µm frozen sections were rinsed in 60% isopropanol and stained with Oil Red O (0.5% in isopropanol) solution for 15 min. Sections were then rinsed with 60% isopropanol and counterstained with hematoxylin. For analysis, multiple images were acquired for consecutive sections and quantitated using ImageJ.

Immunohistochemistry was performed on paraffin sections with the following primary antibodies: CYP1A1 (1:50, sc-101828; Santa Cruz Biotechnology, Dallas, TX, USA), CYP1B1 [1:250 (IF), 1:500 (IHC), Walker et al., 1998], LRIG1 (1:25, AF3688, R&D Systems, Minneapolis, MN, USA) and LGR6 (1:100, sc-393010, Santa Cruz Biotechnology). Prior to incubation in anti-CYP1A1 and anti-LRIG1 antibodies, antigen unmasking was performed by placing sections in citrate buffer (10 mM, pH 5.5) at 85 °C for 6 min. Signals were detected using streptavidin/biotin system (VECTASTAIN^®^ Universal Quick HRP Kit, PK-8800, Vector Laboratories, Burlingame, CA, USA) in combination with diaminobenzidine (DAB substrate kit, SK-4100, Vector Laboratories) per manufacturer’s instructions. The staining intensity for each sample was manually scored on a scale of 0 to 3 for CYP1A1 and 0 to 4 for CYP1B1. A score of 0 was given when no signal was observed, and a score of 3 (in the case of CYP1A1) and a 4 (in the case of CYP1B1) when the highest signal was observed. Fluorescent staining was visualized using appropriate species-specific secondary antibodies conjugated to Alexa Fluor 488, 568, or 647 (1:50, Thermo Fisher Scientific). Slides were mounted using ProLong Diamond antifade reagent containing DAPI as a nuclear counterstain. All fluorescent sections were imaged with a Nikon A1 confocal microscope (Nikon, Melville, NY, USA).

### 2.6. RNA Sample Collection, Processing, and Quantitative Real Time-PCR (qRT-PCR)

A piece of scalp skin tissue was collected in RNA*later* solution (AM7021, Invitrogen, Carlsbad, CA, USA) and stored at −20 °C until processing. For processing, samples were thawed and ~50–100 mg of tissue was homogenized in RNA STAT-60 using a Polytron system (PT-DA 1205/2EC, Kinematica, Switzerland). RNA was extracted with chloroform:isoamyl alcohol (24:1), precipitated with isopropanol, washed with ethanol, and resuspended in water. qRT-PCR was performed as previously described [40] using primers *Cyp1a1* [Forward primer (FP) 5′-GCACCTCTGTTCACCCTACA-3′, reverse complement (RC) 5′-AGACCTGGTTTTACTGCCCA-3′], *Cyp1b1* (FP 5′-TAGTAAGGCTGGGACGGTGA-3′, RC 5′-CATCCGGGTCTGGTTGGTTT-3′), *Artn* (FP 5′-AGCCTTTGCACACTAGACCC-3’, 5′-CTGTTGGTCAGTGGTTCCGA-3’), *Hprt* (FP 5′-ACAGGCCAGACTTTGTTGGA-3′, RC 5′-ACTTGCGCTCATCTTAGGCT-3′), *Il4* (FP 5′-CCCCCAGCTAGTTGTCATCCT-3′, RC 5′-CAAGTGATTTTTGTCGCATCCG-3′) [41]. Expression levels were calculated using individual efficiency values for each primer set and normalization with the reference gene, *Hprt* [42].

### 2.7. Microbiome Analysis

For microbiome sample collection, a sterile foam swab (25-1506 1PF, Puritan, Guilford, ME, USA) moistened with sterile PBS was vigorously rubbed over a 2 × 2 cm area on the dorsal region of the animals. The swab with the microbiome sample was broken off into a sterile tube (2.0 mL Safe-Lock Biopur tubes, 022600044, Eppendorf) and stored at −80 °C until DNA extraction.

### 2.8. DNA Extraction, 16S rRNA Gene Amplicon Library Construction and Sequencing

Bacterial DNA was extracted as described previously [43] using the PureLink kit (K182002, Invitrogen) and quantified using the Quant-iT PicoGreen Assay Kit (P7589, Invitrogen). The 16S ribosomal RNA genes were amplified using bar-coded PCR primers targeted to the V1-V3 hypervariable region (FP 5′-AGAGTTTGATCCTGGCTCAG-3′; RC 5-ATTACCGCGGCTGCTGG-3′). PCR reactions were carried out in quadruplicate using Accuprime Taq HiFi (12346086, Invitrogen). Each PCR reaction contained 0.2 µM of each primer, 1 U AccuPrime Taq HiFi, 1× Buffer II, and 2 µL DNA in a total volume of 25 µL. Cycling conditions are as follows: 1 cycle of 95 °C for 2 min; 32 cycles of 95 °C for 20 s, 60 °C for 30 s, and 72 °C for 60 s; 1 cycle of 72 °C for 5 min. The resulting 16S rDNA amplicons were purified using a 1:1 volume of SPRI beads (09-981-123, GE Healthcare, Chicago, IL, USA), quantified using PicoGreen, pooled in equal amounts, and sequenced on the Illumina MiSeq using 2 × 300 bp chemistry. Extraction blanks and DNA-free water were subjected to the same amplification and purification procedure to assess potential environmental contamination. Library preparation and sequencing were performed at the CHOP Microbiome Center (University of Pennsylvania, Philadelphia, PA, USA).

### 2.9. Bioinformatics Analysis of 16S Amplicon Sequencing Data

Raw FASTQ read files called from Illumina MiSeq machine were demultiplexed using the FLEXBAR program (v2.4) [44] and in-house Perl scripts. All PE-reads of every sample were phylogenetically assigned to the curated Greengenes 97% OTU reference tree (v13.8) [45] using our recently developed phylogenetic placement tool HmmUFOtu [46] with default options. Subsequently, phylogeny-based OTUs and the corresponding OTU-tree were summarized and built using HmmUFOtu with a requirement of a minimum of 5 reads. This cut-off was determined by a rarefaction curve of the remaining number of OTUs.

The OTU table and corresponding tree files were loaded and processed using the phyloseq R package [47]. The taxonomy aggregated summary of microbiome samples, as well as the within-sample alpha-diversity analyses, were also performed using the phyloseq R package. To find differentially enriched OTUs (DE-OTUs), the phyloseq object was first converted into a DESeq2 object, then a negative binomial linear model was trained for the normalized OTU counts using the DESeq2 R package [48], in which both the postnatal age and exposure (corn-oil or TCDD) factors were included. Significant DE-OTUs were called as FDR-adjusted *p* < 0.1. The data presented in this study are openly available in NCBI Sequence Read Archive (SRA) at https://www.ncbi.nlm.nih.gov/bioproject/PRJNA748359 (accessed on 27 July 2021), with a BioProject accession number assignment PRJNA748359.

### 2.10. Serum IgE Detection

Trunk blood for serum IgE detection was clotted (room temperature, 15 min) and then spun for 10 min at 10,000 rpm. Serum was collected as supernatant. Total IgE in the serum was detected by using the Mouse IgE ELISA MAX Deluxe kit (432404, BioLegend, San Diego, CA, USA) according to the manufacturer’s instructions.

### 2.11. Statistical Analysis

Data are presented as the mean values ± SD. A Student’s *t*-test, analysis of variance, and Mann–Whitney U test were used for statistical analysis; *p* < 0.05 was considered statistically significant. All data were analyzed with GraphPad Prism software (version 7.03; GraphPad Software, La Jolla, CA, USA).

## 3. Results

### 3.1. AHR-Dependent Effects of TCDD on the Development of the Epidermal Barrier

**Figure 1:**
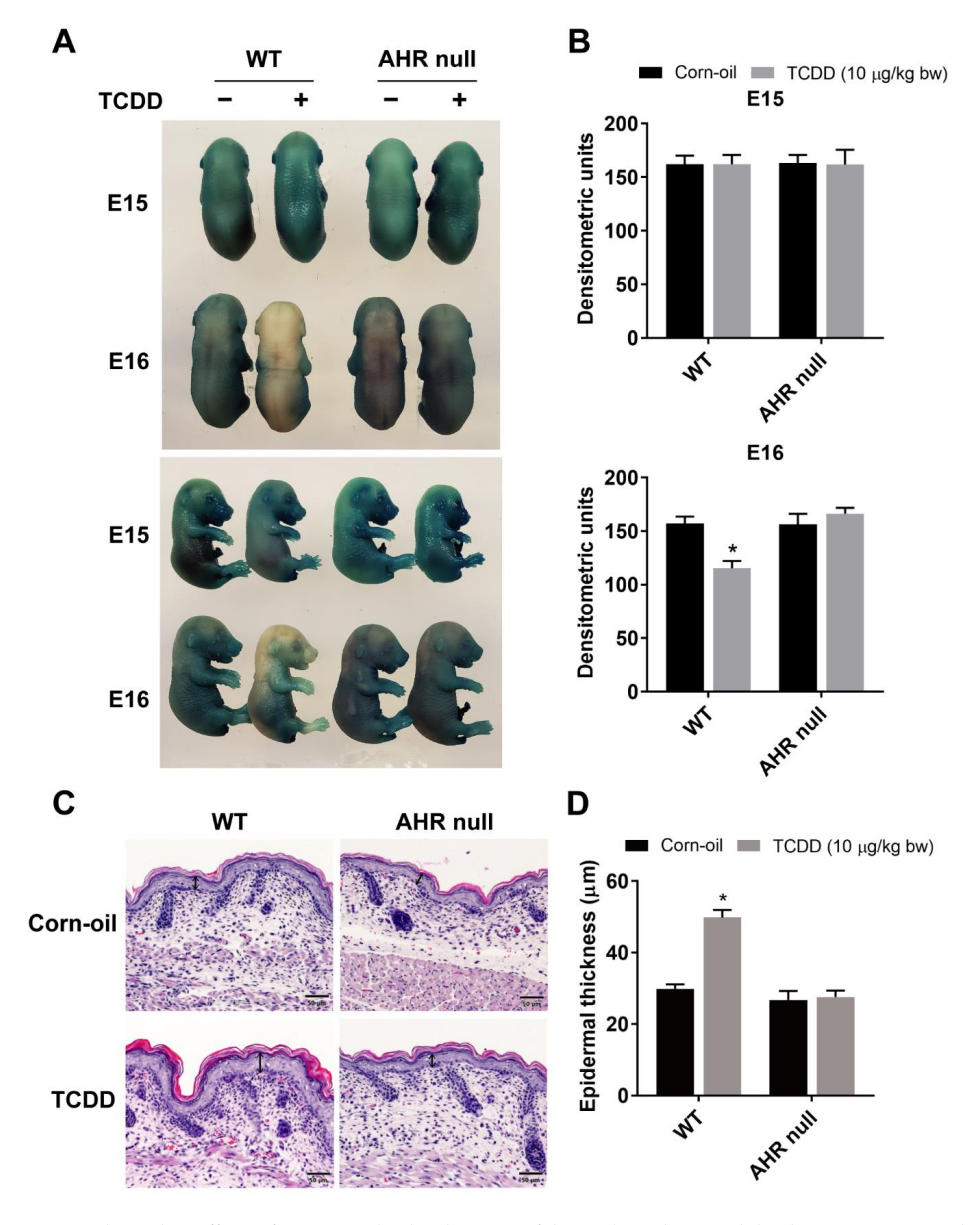
AHR-dependent effects of TCDD on the development of the epidermal permeability barrier. Time-mated mice were treated by gavage with either corn-oil or TCDD (10 μg/kg bw) at E12 or E13 and sacrificed at E15 or E16, respectively (**A**,**B**). (**A**) Representative photographs of fetuses following the X-gal skin permeability assay. (**B**) Quantitation of X-gal staining by densitometry (*n* = 5). (**C**) Representative images of formalin-fixed, paraffin-embedded tissue sections stained with H&E. Time-mated mice were treated by gavage with either corn-oil or TCDD (10 μg/kg bw) at E12 and sacrificed at P1. Scale bar = 50 µm. Double-headed arrows indicate the epidermal thickness. (**D**) Quantitation of epidermal thickness at P1 by microscopy (*n* = 5–6). Bar graphs display the mean values ± SDs. Data were analyzed by two-way ANOVA followed by the Tukey test. * *p* < 0.05, compared to corn-oil control. Abbreviations: AHR, aryl hydrocarbon receptor; TCDD, 2,3,7,8-tetrachlorodibenzo-*p*-dioxin; bw, body weight; E, embryonic day; X-gal, 5-bromo-4-chloro-3-indolyl-β-d-galactopyranoside; H&E, hematoxylin and eosin; P, postnatal day; SD, standard deviation; ANOVA, analysis of variance.

Previously, we reported that in utero exposure to TCDD accelerated epidermal barrier formation in mice [35,36]. To determine whether this effect was dependent on the AHR, *AhR*−/− mice were studied. Time-mated wild type and *AhR* null mice were treated by gavage with TCDD at E12 or E13 and sacrificed at E15 or E16 when an X-gal dye exclusion assay was performed on the fetuses. As previously observed, TCDD accelerated barrier formation of wild-type embryos at E16 compared to corn-oil-treated control pups as shown by the increased exclusion of the dye (Figure 1A,B). This effect was absent in *AhR* null embryos, demonstrating the AHR dependency on the TCDD-mediated acceleration of barrier formation. Further, TCDD treatment induced thickening of the epidermis at P1, indicating acanthosis. This effect was also dependent on the AHR (Figure 1C,D).

### 3.2. Effects of In Utero and Lactational Exposure of Mice to TCDD

**Figure 2:**
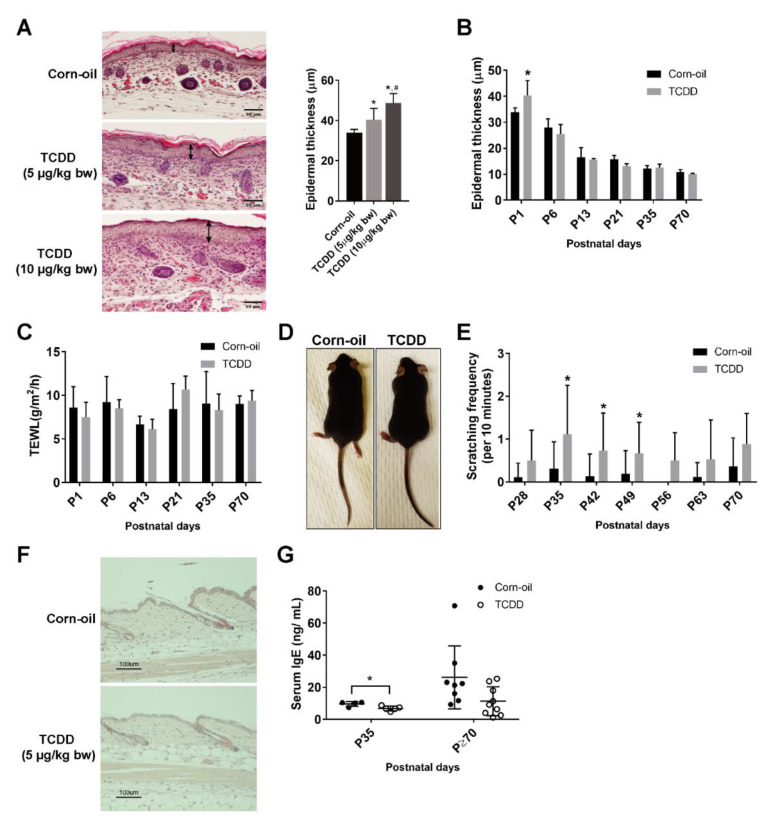
Cutaneous effects of in utero and lactational exposure of mice to TCDD. Time-mated mice were treated by gavage with either corn-oil or TCDD [5 μg/kg bw (**A**–**H**) or 10 μg/kg bw (**A**)] at E12 and pups were examined for cutaneous effects at the indicated postnatal day. (A) Histological analyses of murine pup skin at P1 following the indicated treatments. Representative images of formalin-fixed, paraffin-embedded tissue sections stained with H&E, Scale bar = 50 µm. Double-headed arrows indicate the epidermal thickness. Quantitation of epidermal thickness by microscopy (*n* = 5–7). Bar graph displays the mean values ± SDs. (**B**) Quantitation of epidermal thickness by microscopy at the indicated postnatal day (*n* = 4–6). Bar graph displays the mean values ± SDs. (**C**) Barrier function measured by TEWL (*n* = 6). Bar graph displays the mean values ± SDs. (**D**) Representative image of the appearances of the corn-oil- and TCDD-treated mice at P35 (*n* > 20). (**E**) Frequency of scratching following the indicated treatments at the indicated ages (*n* = 8–15). Bar graph displays the mean values ± SDs. (**F**) Histological analyses of murine pup skin at P70 following the indicated treatments. Representative images of formalin-fixed, paraffin-embedded tissue sections stained with H&E, Scale bar = 100 µm. (**G**) Serum IgE levels in animals at P35 and ≥P70 (between P70 and P135) following the indicated treatments. Each symbol represents an individual animal (*n* = 4–9). Graph displays the mean values ± SDs. Data were analyzed by a two-tailed Student’s *t*-test. * *p* < 0.05, compared to age-matched corn-oil control. ^#^ *p* < 0.05 compared to 5 μg/kg bw TCDD group. Abbreviations: TCDD, 2,3,7,8-tetrachlorodibenzo-*p*-dioxin; bw, body weight; E, embryonic day; P, postnatal day; H&E, hematoxylin and eosin; IgE, immunoglobulin E; TEWL, transepidermal water loss; SD, standard deviation.

No differences in the weights of the control and TCDD-treated mice were observed (data not shown). Pups exposed to TCDD developed epidermal acanthosis at birth. This effect was dose-dependent (Figure 2A). Interestingly, the epidermal thickening observed in the skin of treated mice at P1 did not persist to later postnatal days (Figure 2B). The TEWL values in the treated mice were similar to the control mice, indicative of an undisrupted barrier (Figure 2C). AHR-CA mice exhibit AD-like phenotypes with frequent scratching, dysfunctional barrier, and increased skin inflammation [17]. To determine whether exposure of mice to TCDD in utero and through lactation caused similar skin effects, TCDD-exposed mice were followed from birth to P135. At no time point did the mice display visible signs of skin lesions, including hair matting and loss [17] (Figure 2D). A slight, but significant increase in scratching was observed between P35 and P49 (Figure 2E); however, the frequency (~1 per 10 min) was markedly lower than the frequency observed in the AHR-CA mice (~25 per 10 min) [17]. Inflammation of the skin was also not apparent, as no infiltrating immune cells were visible by H&E staining (Figure 2F). As the inflammatory Th2 cell response is specifically induced in cases of AD, the Th2 cytokines (*Il-4*, *5*, and *13*) were measured, but none were detected in the skin of either control or treated mice (data not shown). There was a slight, but significant, decrease in serum IgE at P35 by TCDD, but no significant difference was observed at P70 or after (Figure 2G). In summary, TCDD-exposed mice showed transient increases in epidermal thickening and minimal signs of inflammation.

### 3.3. Effects of TCDD on Topical MC903-Induced AD-Like Dermatitis

**Figure 3:**
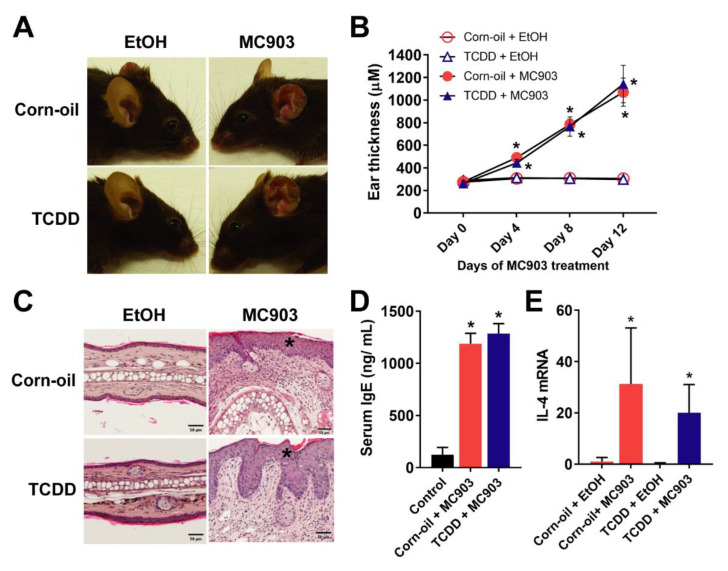
Effects of TCDD on topical MC903-induced AD-like dermatitis. MC903 (2 nmol in 25 µL EtOH) or EtOH (vehicle control) was applied once daily for 14 days to ears of mice exposed to either corn-oil or TCDD (5 µg/kg bw) in utero. (**A**) Representative images of the gross appearance of MC903-treated ears at the end of the treatment. (**B**) Thickness of the ears measured on indicated days of MC903 treatment (*n* = 5). Graph displays the mean values ± SDs. Data were analyzed by two-way ANOVA followed by the Tukey test. * *p* < 0.05, compared to EtOH control for each treatment. (**C**) Representative images of H&E staining of ear sections at day 14. Scale bar = 50 µm, * identifies epidermal hyperplasia. (**D**) Total serum IgE (*n* = 5) at the end of MC903 treatment. Bar graph displays the mean values ± SDs. Data were analyzed by one-way ANOVA followed by the Tukey test. * *p* < 0.05, compared to vehicle control. (**E**) *IL-4* mRNA levels measured by qPCR in skin tissue (*n* = 5) at the end of MC903 treatment. Graph displays the mean values ± SDs. Data were analyzed by two-way ANOVA followed by the Tukey test. * *p* < 0.05, compared to EtOH control for each treatment. Abbreviations: TCDD, 2,3,7,8-tetrachlorodibenzo-*p*-dioxin; AD, atopic dermatitis; EtOH, ethanol; bw, body weight; H&E, hematoxylin and eosin; IgE, immunoglobulin E; IL-4, interleukin 4; qPCR, quantitative PCR; SD, standard deviation; ANOVA, analysis of variance.

Because of the lack of inflammation and the reduction in the levels of IgE in the mice following exposure to TCDD and because TCDD is an immunosuppressant [49], an established model of MC903-induced AD-like dermatitis [50] was used to determine whether the TCDD exposure in our experiments caused long-term immunosuppression. MC903 was topically painted on the ears of in utero corn-oil- and TCDD-exposed mice for 14 consecutive days and resulted in visible scaling and reddening of ears (Figure 3A). MC903-applied ears were significantly thicker and showed inflamed histology with epidermal hyperplasia in both the corn-oil- and TCDD-treated mice (Figure 3B,C). MC903 treatment also induced increased serum IgE levels and *Il4* transcript levels in both the corn-oil- and TCDD-treated animals, indicative of AD-like inflammatory pathology (Figure 3D,E). Levels of *Il4* mRNA appeared to be lower in TCDD + MC903-treated animals compared to the control + MC903-treated animals, but the difference was not significant. Overall, there was no difference in the response to MC903-induced dermatitis between corn-oil- and TCDD-exposed animals.

### 3.4. Biomarkers of Response to TCDD

**Figure 4:**
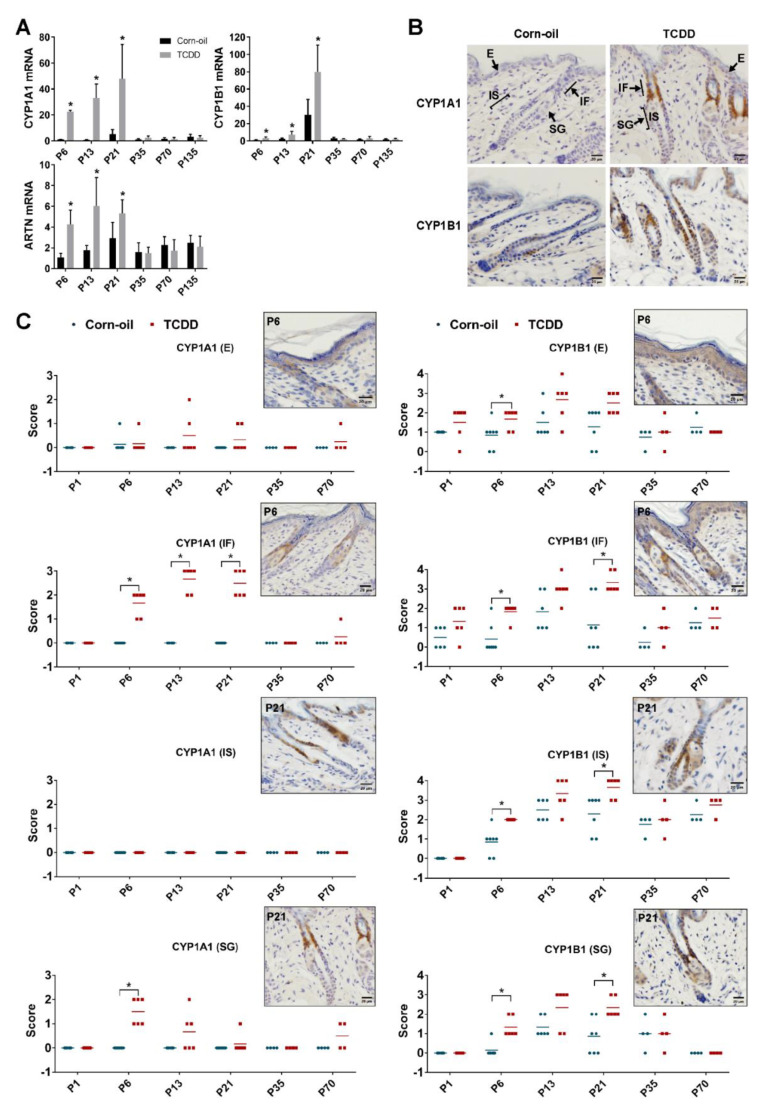
Biomarkers of response to TCDD. Time-mated mice were treated by gavage with either corn-oil or TCDD (5 μg/kg bw) and pups were analyzed for biomarkers of AHR activation. (**A**) Murine skin *Cyp1a1*, *Cyp1b1*, *Artn* mRNA expression measured by qPCR at the indicated age (*n* = 4–6). Data were analyzed by two-tailed Student’s *t*-test. * *p* < 0.05, compared to age-matched corn-oil control. (**B**) Representative images of immunohistochemical staining of CYP1A1 and CYP1B1 at P21 following the indicated treatment. Scale bar = 20 µm. Arrows point towards E, IF, IS, and SG. (**C**) CYP1A1 and CYP1B1 immunostaining intensity scores for corn-oil- and TCDD-treated samples. Staining intensity at different areas of localization was scored manually on a scale of 0 to 3 (CYP1A1) or 0 to 4 (CYP1B1), 0 being absence of staining and 3 or 4 being highest intensity of staining for CYP1A1 or CYP1B1, respectively. Each symbol represents an individual skin sample (*n* = 4–7). Graphs display mean values. Images in boxes are representative immunostaining of TCDD-treated sample at P6 (E, SG) and P21 (IF, IS) as per localization of maximum intensity. Scale bar = 20 µm. Data were analyzed by the Mann–Whitney U test. * *p* < 0.05, compared to age-matched corn-oil control. Abbreviations: TCDD, 2,3,7,8-tetrachlorodibenzo-*p*-dioxin; bw, body weight; AHR, aryl hydrocarbon receptor; CYP1A1, cytochrome P4501A1; CYP1B1, cytochrome P4501B1; ARTN, artemin; qPCR, quantitative PCR; P, postnatal day; E, epidermis; IF, infundibulum; IS, isthmus; SG, sebaceous gland.

To evaluate the toxicodynamics of TCDD in the murine skin, the expression of cytochrome P4501A1 (*Cyp1a1*) and cytochrome P4501B1 (*Cyp1b1*), biomarkers of AHR activation, were examined. Levels of each of these mRNAs were increased in the skin of TCDD-treated animals until P21 and decreased to control levels by P35 (Figure 4A). Immunohistochemical analyses showed that increases in CYP1A1 and CYP1B1 protein levels were highest at P13 and P21 (Figure 4B,C). Further, the cell-specific localization of these proteins was also analyzed. Control CYP1A1 levels throughout the epidermis and pilosebaceous unit were predominantly undetectable. Following TCDD exposure, CYP1A1 was detected in the sebaceous gland and infundibulum, with maximum expression at the infundibulum. In contrast, control CYP1B1 levels increased throughout the epidermis and pilosebaceous unit with the age of the mice. This expression was further increased by TCDD in each of these areas, namely the epidermis, infundibulum, isthmus, and sebaceous gland, with maximum expression detected in the infundibulum and isthmus (Figure 4C). The TCDD-mediated increases in both proteins in all locations decreased to control levels by P35. mRNA expression of *Artn*, a gene linked to alloknesis and pruritus in the AD-like phenotype of the AHR-CA mice [18], was measured. Its expression was increased by TCDD at P6 through P21, but by P35, TCDD-exposed mice did not exhibit increased *Artn* expression (Figure 4A).

### 3.5. Effects of TCDD on Sebaceous Gland Morphogenesis

**Figure 5:**
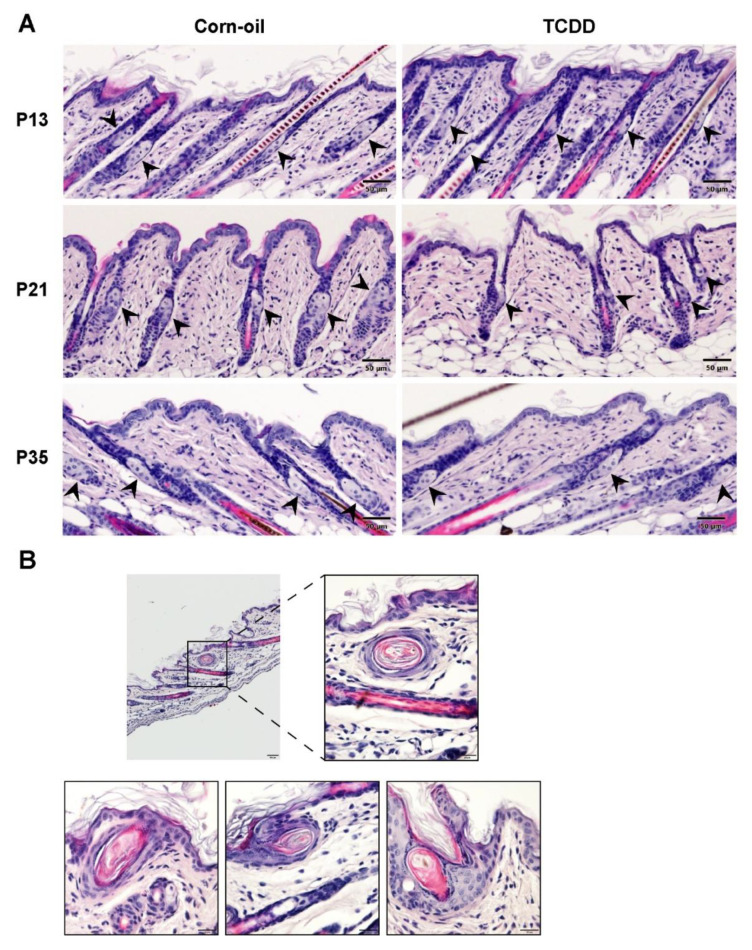
Sebaceous hypoplasia in TCDD-exposed mice. Time-mated mice were treated by gavage with either corn-oil or TCDD (5 μg/kg bw) and pups were analyzed for effects on the pilosebaceous unit. (**A**) Representative images of H&E staining of corn-oil- and TCDD-treated skin at P13, P21, and P35. Scalebar = 50 µm. Arrowheads point towards sebaceous glands. (**B**) Sections of TCDD-treated mice skin stained with H&E at P21 showing the development of keratinous cysts (*n* = 4). Scalebar = 50 µm. Magnified images in boxes, scalebar = 20 µm. Abbreviations: TCDD, 2,3,7,8-tetrachlorodibenzo-*p*-dioxin; bw, body weight; H&E, hematoxylin and eosin; P, postnatal day.

**Figure 6:**
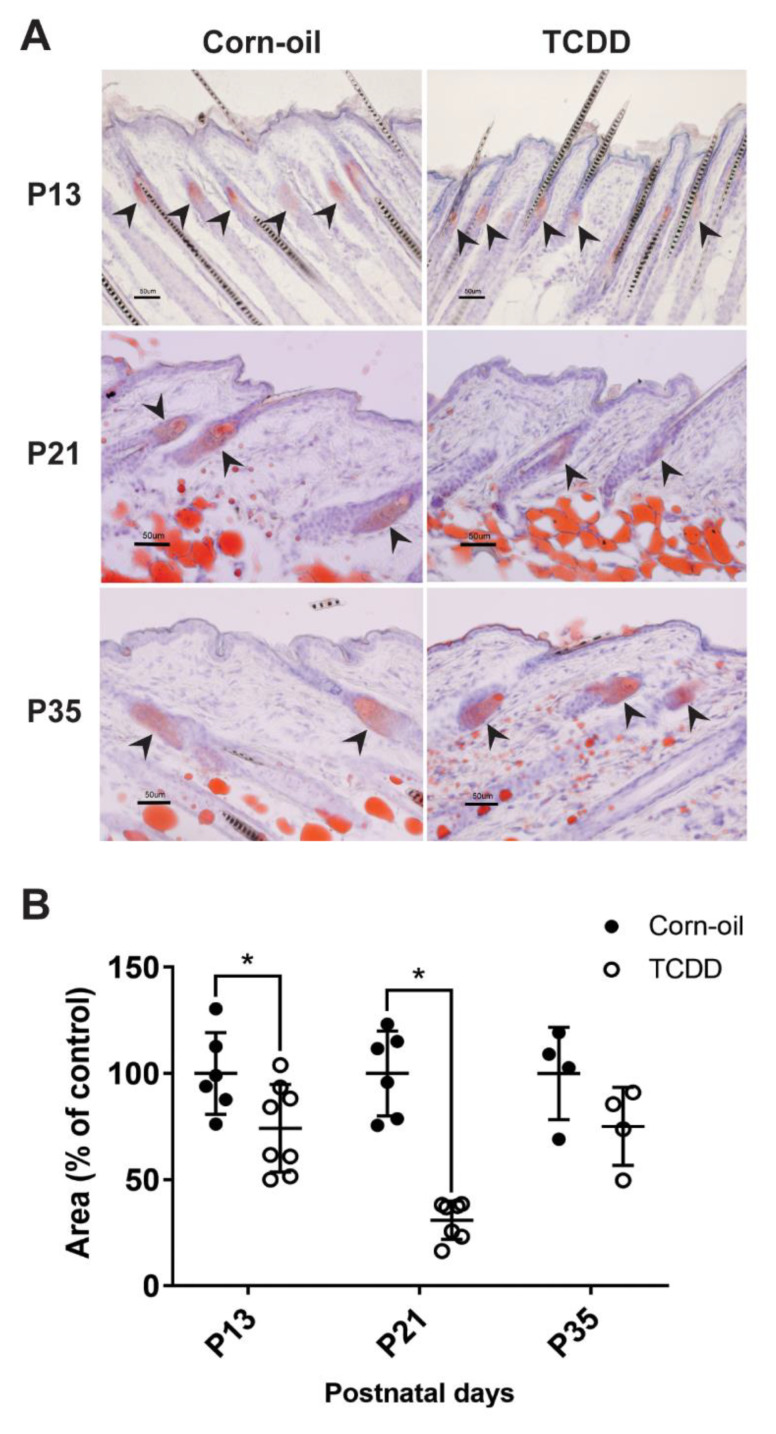
Sebaceous gland morphology and lipid storage. Time-mated mice were treated by gavage with either corn-oil or TCDD (5 μg/kg bw) and pups were analyzed for effects on sebaceous glands. (**A**) Representative images of Oil Red O staining of corn-oil- and TCDD-treated frozen skin sections at P13, 21, and 35. Scalebar = 50 µm. Arrowheads point towards sebaceous glands stained with Oil Red O. (**B**) The quantitation of the area of Oil Red O-stained sebaceous gland is expressed as a percentage of control. The graph displays the mean values ± SDs. Each symbol represents an individual animal (*n* = 4–8). Data were analyzed by a two-tailed Student’s *t*-test. * *p* < 0.05, compared to age-matched corn-oil control. Abbreviations: TCDD, 2,3,7,8-tetrachlorodibenzo-*p*-dioxin; bw, body weight; P, postnatal day.

Chloracne is the hallmark of TCDD toxicity in humans. Cutaneous lesions of chloracne include epidermal thickening, comedones, and metaplastic changes of sebocytes, resulting in atrophy or loss of sebaceous glands. The effects of TCDD on the sebaceous units in exposed murine skin was studied. As observed in the H&E-stained samples, TCDD-treated skin had visibly smaller sebaceous glands compared to control skin indicating sebaceous hypoplasia at P13 and P21 (Figure 5A). This sebaceous gland effect was no longer observed by P35 (Figure 5A). Additionally, four of the eleven TCDD-treated animals developed keratinous cyst-like structures in the epidermis, while none of the control animals developed cysts (Figure 5B). Oil Red O staining of the sebum in the sebaceous glands confirmed that TCDD-exposed samples at P13 had less prominent sebaceous glands compared to the control mice (Figure 6A). The difference was greater at P21 (Figure 6A), where the sebaceous glands in TCDD-treated skin were about 30% of the area of the sebaceous glands in the control skin. By P35, the area of the sebaceous glands in the TCDD-treated mice returned to control levels (Figure 6A,B). Neither other histological cutaneous alterations nor cellular infiltration and inflammation were observed.

### 3.6. TCDD-Responsive Cells in the Pilosebaceous Unit

**Figure 7:**
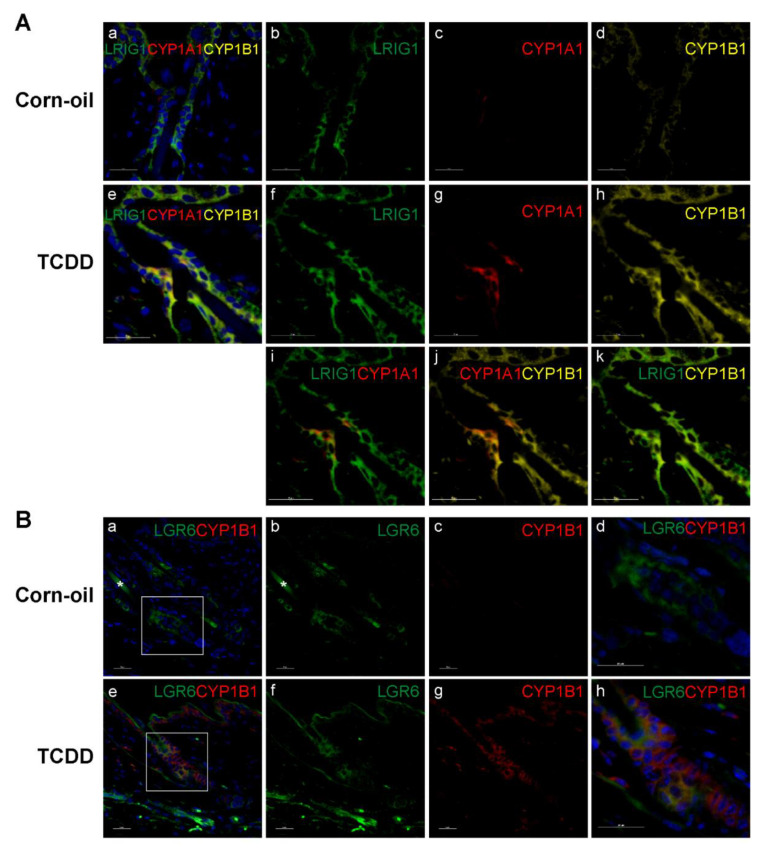
Expression of skin stem cell progenitor markers and AHR biomarkers in skin. Time-mated mice were treated by gavage with either corn-oil or TCDD (5 μg/kg bw) and pups were analyzed for specific localization of response to TCDD. Corn-oil- or TCDD-exposed skin samples at P21 were labeled with antibodies as indicated. DAPI (blue) was used to stain nuclei. (**A**) CYP1A1, CYP1B1, and LRIG1 expression in corn-oil- (**a**–**d**) and TCDD- (**e**–**k**) treated skin samples, as indicated. (**B**) CYP1B1 and LGR6 expression in corn-oil- (**a**–**d**) and TCDD- (**e**–**h**) treated skin, as indicated. Boxed areas in (**a**,**e**) are shown at higher magnification in (**d**,**h**), respectively. Scalebar = 25 µm. Abbreviations: AHR, aryl hydrocarbon receptor; TCDD, 2,3,7,8-tetrachlorodibenzo-*p*-dioxin; bw, body weight; P, postnatal day; DAPI, 4′,6-diamidino-2-phenylindole; CYP1A1, cytochrome P4501A1; CYP1B1, cytochrome P4501B1; LRIG1, Leucine-rich repeats and immunoglobulin-like domains protein 1; LGR6, Leucine-Rich Repeat Containing G Protein-Coupled Receptor 6.

Sebaceous glands are maintained by a population of stem and progenitor cells that reside in specific niches of the hair follicles. To determine if these cells are possible targets of TCDD, skin samples at P21 were immunostained with CYP1A1, CYP1B1, and with markers of sebocyte progenitor cells, LRIG1 and LGR6. LRIG1 is a marker of sebocyte progenitor cells reported to be located at the infundibulum and junctional zone [51]. Following TCDD exposure, the expression of CYP1A1 increased at the infundibulum and junctional zone (Figure 4B,C and Figure 7A(c,g)) and colocalized with the expression of LRIG1 (Figure 7A(i)). The expression of CYP1B1 was also induced by TCDD. Increased expression was observed throughout the epidermis and pilosebaceous unit (Figure 4B,C and Figure 7A(d,h)) and colocalized with the expression of LRIG1 and CYP1A1 (Figure 7A(k,j), respectively). LGR6 is a well-established marker of sebocyte progenitor cells that has been identified in the isthmus, bulge, sebaceous glands, and epidermis [52,53,54,55]. LGR6 expression was observed in the upper and lower isthmus (Figure 7B(b,f)) and the expression of CYP1B1 colocalized at these locations in the TCDD-exposed skin (Figure 7B(h)).

### 3.7. Effects of TCDD on Microbiome Assembly

**Figure 8:**
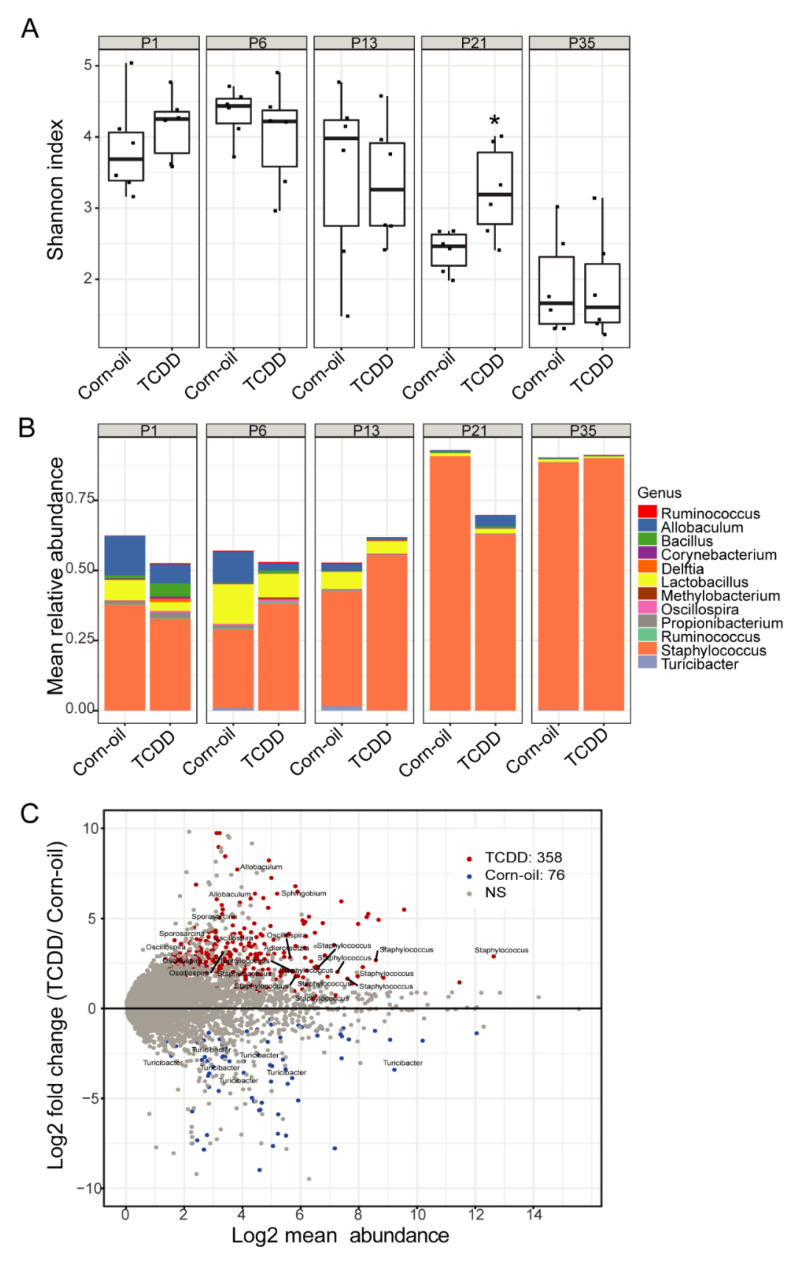
TCDD effects on murine skin microbiota revealed by 16S rRNA gene sequencing. Time-mated mice were treated by gavage with either corn-oil or TCDD (5 μg/kg bw) and pups were analyzed for effects on the skin microbiome. (**A**) Alpha diversity of skin microbiota as measured by Shannon index (y-axis) following the indicated treatments and time. Data are displayed as box plots. Each symbol represents an individual animal (*n* = 6). Data were analyzed by the Wilcoxon rank sum test. * *p* < 0.05, compared to age-matched corn-oil control. (**B**) Stacked bar plot of mean relative abundance (y-axis) of the top 12 genera identified in the skin microbiota following the indicated treatments and time. (**C**) MA-plot comparing differentially enriched OTUs between corn-oil- or TCDD-treated skin microbiota at P21. Shown is log2 mean abundance (x-axis) and log2 fold-change of normalized OTU counts (y-axis). Color of points indicates whether OTUs were significantly increased in TCDD (red), corn-oil (blue), or not significant (NS; grey). The top 30 most significant OTUs that have a genus-level taxonomy assignment are highlighted. Abbreviations: TCDD, 2,3,7,8-tetrachlorodibenzo-*p*-dioxin; bw, body weight; P, postnatal day; OTU, operational taxonomic unit.

Skin is a habitat to a diverse community of microorganisms that contribute to the establishment of a protective barrier to prevent invasion by other pathogens [56]. To determine the effects of AHR activation by TCDD on the assembly and shifts of the skin microbiome during development, 16s rRNA gene sequencing was performed on the microbiome isolated from skin swabs. The Shannon diversity index was determined to evaluate the overall microbial community across treatments and time points. Compared to corn-oil-treated skin, TCDD-exposed skin exhibited significantly increased bacterial diversity at P21. No significant shifts in bacterial community were observed between the treatment groups at any other time point (Figure 8A). Comparing the relative abundance of the most prominent taxa in each treatment group, *Staphylococcus* represented the most abundant genera across all time points in both the control and treatment groups (Figure 8B). Further, at P21, the relative abundance of *Staphylococcus* was reduced and that of *Allobaculum* was increased in TCDD-treated samples as compared to corn-oil-treated samples (Figure 8B). Finally, the differential abundance analysis identified 358 OTUs as significantly abundant in TCDD-treated samples and 76 OTUs that were significantly abundant in corn-oil-treated samples, including taxa of *Allobaculum* and *Sphingobium* that were enriched in TCDD-exposed mice at P21 (Figure 8C).

## 4. Discussion

Recently, Hidaka and others reported that the constitutively active expression of the AhR in murine keratinocytes causes the development of an AD-like phenotype consisting of allergic inflammation, pruritis, and epidermal barrier disruption [18]. Our previous work on the developmental effects of TCDD on skin function demonstrates that in utero exposure to TCDD causes acanthosis, epidermal hyperkeratosis, and an abnormal epidermal permeability barrier with leaky tight junctions in the pups at birth [36]. This impairment of the epidermal barrier by TCDD suggested to us that in utero exposure to AHR-activating pollutants might be an important factor in the development of AD in children.

The prevalence of AD in children is high and increasing worldwide. Although the reasons for this increase are not well understood several factors including environmental-gene interactions are proposed [57]. Human exposure to environmental pollutants such as TCDD occurs through multiple routes including lactation [58]. To determine if developmental exposure to TCDD resulted in adverse inflammatory murine skin effects after birth, similar to the AHR-CA mice, embryos and pups were exposed in utero and during lactation and analyzed from E15 through P135. Importantly, this mode of exposure mimics the transfer of lipophilic bioaccumulated environmental pollutants from mother to offspring in humans.

Here, exposure to TCDD caused an increase in pruritis after birth. This increase was transient and much lower compared to the pruritis observed in the AHR-CA mice. We did not detect a change in the skin TEWL, nor an increase in cutaneous inflammation, indicating that systemic ligand activation of the murine AHR via in utero and lactational exposure does not cause an inflammatory skin disease as found in the AHR-CA mice. Moreover, IgE levels were slightly lower in the exposed pups at P35 and no difference was observed at later times indicating a lack of allergic sensitization.

In order to better understand the lack of inflammation and minimal increase in pruritis following in utero and lactation exposure to the AHR ligand, TCDD, we carried out several studies. To investigate if systemic in utero and lactational exposure to TCDD caused immunosuppression and possibly contributed to a lack of an inflammatory response in our study, a model of MC903-mediated Th2-like inflammation was used. The severity of the response to MC903 was not different in the control and TCDD-exposed animals, indicating that long-term immunosuppression via systemic exposure to TCDD did not affect the inflammatory response. Additionally, to determine the time course of response to TCDD, the expression of sensitive biomarkers of AHR activation, *Cyp1a1*, and *Cyp1b1*, were measured. Both RNA and protein levels were increased until P21, after which they returned to control levels. This was also true for *Artn*, a neurotrophic factor involved in the itching/scratching behavior that contributes to a disrupted barrier in the AHR-CA mice. ARTN is an axon-guiding protein involved in epidermal hyper-innervation, linked to alloknesis. Antibody neutralization of ARTN expression decreases epidermal hyper-innervation and alloknesis in the AHR-CA mice. Alloknesis-induced scratching is responsible for barrier damage that leads to enhanced antigen penetration and sensitivity to antigens and an inflammatory response [18]. Here, *Artn* expression was increased in the skin of pups exposed to TCDD, but the increases were not near the same magnitude as reported in the AHR-CA mice and were not persistent, decreasing to the control level after P21, similar to *Cyp1a1* and *Cyp1b1*. The response of these biomarkers and the levels of *Artn* expression are consistent with the much lower scratching frequency and lack of inflammation observed in the TCDD-exposed pups. Transient activation of the AHR is likely due to the relatively short half-life (11 days) of TCDD in mice [59] and indicates that sustained activation of the AHR may be necessary for exposed pups to develop more severe skin abnormalities. As the expression of ARTN is a critical factor in the atopic disease of the AHR-CA mice, we suggest that ligands that do not maintain a sustained and large increase in the level of ARTN may not cause atopic disease. The shorter half-life of TCDD in mice, combined with a short window of exposure in this model (weaning occurs at P21), prevents prolonged exposure to TCDD. While persistent activation of the AHR appears to be necessary to elicit adverse skin outcomes, other effects of in utero and lactational exposures, such as T-cell impairment, are maintained long after TCDD is cleared and have been attributed to persistent changes in DNA methylation [60,61]. Additionally, it should be noted that the half-life of TCDD in humans is significantly longer (7–10 years) compared to the murine half-life (11 days) [62], indicating that the possible reversal of certain human effects from exposure to TCDD or DLCs would be much slower.

Although the effects of gestational and lactational exposure to TCDD did not resemble the inflammatory skin effects seen in the AHR-CA, the observed sebaceous gland atrophy had strong concordance with reports of seboatrophy from topical TCDD exposures of newborn and adult mice for 2 or 5 weeks [16,63]. The TCDD-mediated sebaceous gland atrophy was maximal at P21 and recovered at later time points, consistent with the timing of expression of the AHR biomarkers, CYP1A1 and CYP1B1. As suggested by Fontao et al., the observed atrophy of sebaceous glands indicated that specific progenitor cells that normally differentiate to form the gland have enhanced sensitivity to TCDD. During homeostasis, sebaceous glands are maintained by progenitor cells harbored in specific niches of hair follicles. LRIG1+ progenitor cells localize to the infundibulum and junctional zone and LGR6+ progenitor cells to the isthmus [51,52]. Confirming the previous report by Fontao et al., CYP1A1 expression was detected at the infundibulum and junctional zone, and its expression colocalized with LRIG1+ cells [16]. In contrast, the AHR target gene CYP1B1 [64,65] was detected throughout the hair follicle, sebaceous gland, and epidermis and thus colocalized with LRIG1+ cells as well. This widespread induction of CYP1B1 indicates that TCDD is activating the AHR throughout the epidermis, not just in the infundibulum and junctional zone where CYP1A1 is induced. Additionally, the colocalization of inducible CYP1B1 with LGR6 at the isthmus indicates that not only the LRIG1+ but also LGR6+ progenitor cells are targets of AHR activation by TCDD in the skin. It is unclear why the expression of one AHR gene target, CYP1A1, is restricted to the infundibulum and junctional zone, while a second target, CYP1B1, is not. In sebocyte cell culture, knockdown of LRIG1 decreases TCDD-induced CYP1A1 levels, indicating that LRIG1 promotes the expression CYP1A1 [16].

The AHR is involved in pathways critical to cell cycle, differentiation, and apoptosis [66]. Thus, stem cells that require a balance between self-renewal and differentiation are sensitive targets of AHR. B lymphocyte-induced maturation protein 1 (BLIMP1), a MYC repressor and a marker of sebocyte precursor cells, maintains sebaceous homeostasis. Either *Blimp1* deletion or *Myc* overexpression results in enlarged sebaceous glands [67,68,69]. Lineage tracing studies in mice have shown that BLIMP1+ sebocytes are the progeny of the LRIG1+ and the LGR6+ progenitor cells [70,71]. Interestingly, BLIMP1 co-localizes with the AHR in mouse skin and in the human sebocyte cell line, SZ95, *BLIMP1* expression is induced by activation of the AHR and reduced by AHR knockdown [72]. Furthermore, TCDD treatment induces the atrophy of SZ95 sebocytes [73]. Thus, alteration of BLIMP1 and MYC signaling by AHR activation in LRIG1+ and LGR6+ sebaceous progenitor cells may cause the observed sebaceous atrophy. Clarification of the role of AHR activation in the commitment to differentiation of these progenitor cells would improve the understanding of the molecular mechanisms underlying chloracne.

Interestingly, AHR- and dose-dependent acanthosis was detected throughout the TCDD-exposed population at P1 and keratinaceous cysts were observed in the skin of 4 of 11 TCDD-exposed mice at P21. Acanthosis and cystic lesions in mice have only previously been observed following a 2-year gavage study using 3,3′,4,4′-tetrachlorazobenzene [15]. Until now, shorter-term models to investigate the relationship between sebaceous gland hypotrophy and comedone cyst formation, the hallmarks of DLCs toxicity, have not existed. The sensitivity of developing pups appears to contribute, at least in part, to the manifestation of these conditions.

Also unique to this study, in utero and lactational exposure to TCDD increased the diversity of the skin microbiome. The AHR is now recognized as a major signaling pathway by which the commensal microbiota regulate skin barrier function and repair [74]. The concordant timing of the effects of TCDD exposure to disrupt the pilosebaceous unit and microbial community structure in the skin supports the role of the AHR in the development and maintenance of this system-level interaction between host and microbiome. This effect on the microbial community structure was further supported by the observed changes in the mean relative abundance of the prominent genera identified in the skin microbiome at P21. Among the OTU that were significantly increased at P21 were the taxa of *Allobaculum* and *Sphingobium. Allobaculum* produce short-chain fatty acids with beneficial immunological and metabolic effects [75]. *Sphingobium* degrade a wide variety of polycyclic aromatic hydrocarbons and have been investigated for use in bioremediation [76,77]. Although *Sphingobium* is not known to include a species that specifically metabolizes TCDD, a related genus, *Sphingomonas*, contains a species, *Sphingomonas wittichii* RW1, that uses chlorinated dioxins, including TCDD, as its sole source of carbon for energy and growth [78]. While the observed TCDD-mediated changes to the microbial community correlate with the epidermal changes observed at P21, the relationship between these changes is not yet understood. The structure and function of the skin is dependent on its microbiome and vice versa [79]. Thus, the effects of TCDD on the structure and function of the skin may alter the growth characteristics and life cycle of microbiota. Additionally, TCDD may have direct effects on the cutaneous microbiome and its metabolic capacity, as reported by others for the gut microbiome [80], which could influence epidermal structure and function. Overall, the influence of DLCs on the skin microbiome is understudied but reports on the gut microbiome in mice have shown that DLC exposure results in dysbiotic gut microbiota and alterations in microbiota-host metabolic homeostasis [81,82,83].

## 5. Conclusions

In summary, we did not observe adverse inflammatory skin effects following in utero and lactational exposure to TCDD; we did observe TCDD-mediated acanthosis, sebaceous gland atrophy, and comedone cyst formation, all of which are characteristics of the human condition of chloracne. Thus, this sensitive murine model will be useful in understanding the mechanisms involved in the etiology of this human condition that has been difficult to model in rodents, while also providing new insights into the development, maintenance, and function of the sebaceous gland and its contributions to the skin microbiome.

## Data Availability

The data presented in the murine skin microbiota study are openly available in NCBI Sequence Read Archive (SRA) at https://www.ncbi.nlm.nih.gov/bioproject/PRJNA748359 (accessed on 27 July 2021), with a BioProject accession number assignment PRJNA748359.
